# Adipose Tissue-Endothelial Cell Interactions in Obesity-Induced Endothelial Dysfunction

**DOI:** 10.3389/fcvm.2021.681581

**Published:** 2021-07-01

**Authors:** Manna Li, Ming Qian, Kathy Kyler, Jian Xu

**Affiliations:** ^1^Department of Medicine, Harold Hamm Diabetes Center, University of Oklahoma Health Sciences Center, Oklahoma, OK, United States; ^2^Office of Research Administration, University of Oklahoma Health Sciences Center, Oklahoma, OK, United States

**Keywords:** adipose tissue, endothelial dysfuction, obesity, cell interaction, cardiovascular disease

## Abstract

Obesity has a strong impact on the pathogenesis of cardiovascular disease, which raises enthusiasm to understand how excess adiposity causes vascular injury. Adipose tissue is an essential regulator of cardiovascular system through its endocrine and paracrine bioactive products. Obesity induces endothelial dysfunction, which often precedes and leads to the development of cardiovascular diseases. Connecting adipose tissue-endothelial cell interplay to endothelial dysfunction may help us to better understand obesity-induced cardiovascular disease. This Mini Review discussed (1) the general interactions and obesity-induced endothelial dysfunction, (2) potential targets, and (3) the outstanding questions for future research.

## Introduction

The increased incidence of obesity contributes to the prevalence of various metabolic diseases. About 1.9 billion people are predicted to be obese or overweight, worldwide (1). Obesity is an established risk factor for cardiovascular disease (CVD) (2); obese individuals are predisposed to a range of cardiometabolic abnormalities (3). Thus, great attention has been drawn to the topic of how excess adiposity leads to vascular dysfunction. Mechanistically, adipose tissue (AT) affects the cardiovascular system through the secretion of bioactive products (e.g., adipocytokines and microvesicles), inorganic molecules, and reactive oxygen species (ROS). AT becomes dysfunctional in obesity and generates a pro-inflammatory, hyperlipidemic, and insulin-resistant environment, which ultimately leads to the development of metabolic complications (e.g., diabetes) and cardiovascular complications (e.g., atherosclerosis) (4). Arteries residing in visceral adipose display impaired vascular responses to endothelium-dependent agonists (e.g., intraluminal flow), characteristic of endothelial dysfunction (ED), which occurs long before CVD has developed. Recognizing the roles of AT in obesity-induced ED/CVD, this mini review will discuss the AT-EC interactions with their potential as therapeutic targets of obesity-induced ED and the remaining research questions that merit further investigation.

### The Role of AT in Obesity

AT is the body's largest endocrine organ and secretes hormones, cytokines, and proteins in endocrine and/or paracrine manners that affect cell and tissue function throughout the body (5). AT is also essential in maintaining lipid and glucose homeostasis, which becomes dysfunctional in obesity and excessive deposition of fat occurs. Enlarged adipocytes in obese individuals promoted macrophage-mediated inflammation and adipokine-induced insulin resistance (6). The pathological function of AT is determined by their cellular composition, secretome (secretion profiles), and location in the human body (7). Further, low storage and removal of adipose triglycerides promote dyslipidemia, while high storage and low removal prompt obesity (8). In addition, detection of brown and/or beige AT in adult humans (9, 10) and the realization of adipocyte heterogeneity and plasticity of white AT spurred great interest in targeting AT for possible therapeutic advantages (11).

### Obesity-Induced Endothelial Dysfunction (ED)

Impairment of flow-induced vasodilation, arterial dilation prompted by blood flow, is one hallmark of obesity-induced ED. It is demonstrated by the impairment of endothelial nitric oxide synthase (eNOS) and the loss of nitric oxide (NO), a major vasodilator and anti-inflammatory agent (12). Obesity also promotes damage of the endothelial glycocalyx, which responds to mechanical force from blood vessels and regulates NO production. Flow-mediated vasodilation in mouse and human mesenteric arteries was hindered by loss of the endothelial flow-sensitivity of Kir (inwardly rectifying K^+^) channel due to obesity-induced glycocalyx thinning (13). Defective physiological properties of endothelial cells (EC) will switch the vascular endothelium to a pro-inflammatory, prothrombotic and proatherogenic phenotype, leading to leukocyte adhesion, activation of platelets, and pro-oxidation of mitogens, along with impaired endothelial NO production, decreased synthesis of endothelium-derived hyperpolarizing factors (EDHF), and increased vasoconstriction factors, such as angiotensin II (Ang II) and prostaglandin (PGH2) (14). Through activation of adhesion molecules, leukocyte proliferation, and transmigration, ED reportedly launches CVD progression in obesity (15). Moreover, secretion of angiotensinogen of the renin-angiotensin system (RAS) by dysfunctional adipocytes leads to its overexpression in RAS, enhancing ROS production and increasing the atherogenic and thromboembolic potentials of EC (16). The risks associated with cardiovascular complications can be mitigated through inhibition of inflammatory mechanisms and controlling obesity (16).

## Modes of AT-EC Interaction in Obesity

Human AT can be broadly divided into subcutaneous AT (SAT) and visceral AT (VAT). VAT in the heart can be classified as epicardial AT (EAT) and pericardial AT (PAT). Among all AT depots, the perivascular AT (PVAT) is recognized as a vital regulator of vascular biology because of its anatomical proximity to the vessels (17). These ATs regulate cardiovascular system through the secretion of bioactive products, such as adipokines, microvesicles, and gaseous messengers. The secretome is under tight control by homeostatic mechanisms, which can become dysregulated in obesity. There are two modes of AT-EC interaction: the endocrine mode, which is an indirect crosstalk through the circulation, and the paracrine mode, which is a direct interplay. Obesity-initiated systemic or local inflammation and insulin resistance shift the AT secretome from an anti-inflammatory and anti-atherogenic state toward a pro-inflammatory and pro-atherogenic state.

### Endocrine Mode

Associated with connective tissue and blood vessel proliferation, inflammation has been regarded as the first stage of vascular dysfunction. AT-derived tumor necrotic factor-α (TNF-α) is one product of inflammation (18). In obesity, microvasculature from VAT is an important source of low-grade inflammation and oxidative stress. Both contribute to vascular changes and favor increased atherosclerosis under clinical conditions. Mechanistically, excessive macronutrients accumulated on the AT promote the secretion and release of inflammatory mediators, including interleukin-6 (IL-6), interleukin-1β (IL-1β), TNF-α, leptin, and stimulation of monocyte chemoattractant protein-1 (MCP-1), which subsequently produce less adiponectin, thereby initiating a proinflammatory state (16) and driving vascular destabilization and leakage (19).

Among AT-secreted factors, MicroRNAs (miRNAs) are short, single-stranded, non-coding RNA molecules that play important roles in a variety of cellular processes, such as differentiation, proliferation, apoptosis, and stress response; their alteration contributes to the development of many pathologies, including obesity (20). Specifically, AT-derived miRNA (21) mediates obesity-induced ED by affecting gene expression of eNOS, SIRT1, cellular producers of ROS, autophagy machinery, and ER stress (22).

### Paracrine Mode

PVAT and EAT elicit direct impacts on the adjacent vascular wall or myocardium, respectively, through the paracrine release of bioactive mediators. These mediators travel to neighboring vessels, thereby regulating the biology of entire vascular beds in a “vasocrine” manner (23). In obesity, PVAT-secreted high-concentrations of adipokines (e.g., TNF-α and IL-6) access the vascular lumen and suppress the PI3-K pathway of insulin signaling, which unlocks the vasoconstrictor effects of endothelin 1, leading to a reduction in insulin-mediated muscle nutritive blood flow, contributing to insulin resistance (23). PVAT also releases miRNA, including miR-221-3p, which is highly enriched in obese PVAT and recently reported to induce ED by vascular remodeling (24). EAT has close proximity to the adventitia of the coronary arteries and shares the same microcirculation as the underlying myocardium (25, 26). When adversely remodeled and dysfunctional in obesity, EAT secretes proinflammatory cytokines, contributing directly to the pathogenesis of coronary artery disease (27).

## Targeting AT-EC Axis to Treat Obesity-Induced ED/CVD

AT has an essential role in obesity-induced CVD (28) Recent findings suggest that targeting either AT, EC (single-target), or both (dual-target) with a mechanism-based approach would improve obesity-induced ED/CVD (Table 1).

**Table 1:** Recent advances in treating obesity-induced ED.

**Potential targets/interventions**		**Effects and mechanisms**
Single-target	EC (29)	
	eNOS	Slow-release eNOS substrate arginine (30) or blocking arginase (31, 32) to improve eNOS function and/or NO bioavailability in rodents and patients.
	FTO inhibition	Overcame glucose intolerance and insulin resistance and hypertension in mouse models of obesity (33).
	NOX inhibition	Inhibition of specific subunits ameliorated ROS-induced ED in rat model of obesity (34, 35).
	CD40L inhibition	Improved ROS-induced inflammation and ED in mouse models of obesity (36).
	NETs	Blocking formation or increased degradation in EC prevented ED in mouse model of obesity (37, 38).
	TRPV4 channels	Activity rescue improved ED (39), involving Ca^2+^-mediated vasoregulation (40), in mouse models of obesity.
	Soluble (pro)renin receptor inhibition	Soluble (pro)renin receptor induced ED and hypertension by activating AT1R leading to RAS hyperactivity in mouse models of obesity (41).
	AT (4)	
	GRK2 inhibition	Reduced AT-macrophage infiltration and improved ED in mice (42).
	Anti-inflammation	Reduced AT-pro-inflammatory cytokine production by adipokine and leptin (43).
Dual-target	GLP1 agonist DPP4 inhibitors	Improved cardiovascular outcomes in patients of type 2 diabetes mellitus (44, 45) related to improved AT function (46).
	SGLT2 inhibitor	Cardioprotective (47) and beneficial for heart failure in patients (48).
	Anti-inflammation	Reduced AT-pro-inflammatory cytokine production and restored endothelial function by metformin (49), resveratrol (50), and methotrexate (51).
	Lifestyle intervention	Exercise improved EC function (52) or reversed ED (53). Calorie restriction improved vascular insulin sensitivity and reduced inflammation (54).

### Single-Target Interventions

Targeting peripheral vascular EC to improve ED has been long exploited (29). Recent studies showed that heterozygous eNOS deficiency was associated with ED in diet-induced obesity (55). Patients with abnormal arginine metabolism and bioavailability due to obesity displayed lower cardiometabolic risk after treatment with a slow-release eNOS substrate, arginine (30). Vascular function could be rescued in obese vessels by targeting EC-Ca^2+^ toolkit, although this was not tested in obese subjects (56). A recent study showed that deletion of fat mass and obesity-associated protein (FTO) in EC rescued metabolic and vascular function in obesity (33), independent of its known function in regulation of obesity (57). Vascular arginase reduces NO bioavailability, which hastens microvascular remodeling in obesity (31). EC-derived arginase mediates obesity-induced vascular dysfunction and arterial stiffening (32), implicating arginase as a potential target of obesity-induced ED.

Oxidative stress is a key pathogenic factor of microvascular complications in metabolic disease. Renal Nox1, Nox2, and Nox4 contribute differentially to vascular oxidative stress-associated ED in obesity, suggesting a need to identify specific Nox subunits as a target (34), which may result in effective prevention of obesity-related CVD (35). Oxidative stress-associated inflammation is another frequently tested target. CD40 ligand (CD40L) signaling regulates ED via immune cell recruitment and platelet activation in mouse models of hypertension, the mechanism of which extended to mouse models of obesity, implicating CD40L as a therapeutic target for lipid dysmetabolism (36). Neutrophil extracellular traps (NETs) have an inflammatory web-like chromatin structure (37). Inhibition or degradation of NETs prevented ED in mouse model of obesity (38) Further, adipose macrophage infiltration enhanced vascular ED in obese subjects (58). Blocking infiltration of macrophages and T lymphocytes in PVAT prevented obesity-induced ED in mice with G protein-coupled receptor kinase 2 (GRK2) deletion in myeloid cells (42), suggesting GRK2 as a potential therapeutic target. Deletion of lipoxin receptor in leukocytes led to unsolved inflammation in mice, which augmented ED with diabetic cardiomyopathy in obesity (59). Similar therapeutic potentials have been found in other inflammatory mediators, such as adipokine and leptin (43).

Obesity is a strong predictor of hypertension, although it remains unknown how obesity increases blood pressure (60). ED is a hallmark of obesity-induced hypertension. Insulin resistance and increased systolic blood pressure led to ED in obesity; however, targeting these factors presented with different benefits depending on sex (61) and ethnic group (62). RAS hyperactivity was often thought to result from Ang II-dependent stimulation of the Ang II type 1 receptor (AT1R). Recently, the soluble (pro)renin receptor was found to induce ED and hypertension by activating AT1R in high-fat diet (HFD) feeding mice (41). Another recent study reported that endothelial transient receptor potential vanilloid 4 channels (TRPV4) was impaired in a mouse model of diet-induced obesity and obese human resistance vessels, resulting in increased blood pressure (39). This contrasted with findings from another recent study in the same mouse model, but with a longer duration on HFD feeding, which implicated Ca^2+^-spark vasoregulation as the underlying mechanism (40). Prolonged HFD feeding in mice appeared to improve the vascular response to leptin, which overrode ED induction (63) In any case, strategies to preserve or protect a functional target on EC would bear promise to improve ED and hypertension in obesity.

### Dual-Target Interventions

In terms of safety, cost, and effectiveness, most clinical approaches targeting AT have not been successful in the treatment of AT-induced CVD (28). However, some commonly used anti-hyperglycemic medications and lifestyle intervention could elicit a dual action: improving AT function and conferring an appreciable cardiovascular benefit.

Glucagon-like peptide 1 (GLP1) is an incretin, responsible for insulin secretion, glucagon inhibition, and decreased gastrointestinal motility in the post-prandial setting. GLP1 is inactivated by dipeptidyl peptidase 4 (DPP4), an AT-expressing enzyme. GLP1 agonists (e.g., liraglutide) and DPP4 inhibitors (e.g., sitagliptin) are now being used in the management of type 2 diabetes mellitus with improved cardiovascular outcomes in clinical trials (44, 45), implicating AT involvement (46). Sodium–glucose transporter 2 (SGLT2) (e.g., empagliflozin) is responsible for renal glucose reabsorption, the inhibition of which also exerted direct AT effects with a cardioprotective profile (47). As reported in a meta-analysis, SGLT2 inhibitors generated consistent beneficial outcomes for heart failure and kidney disease, with certain heterogeneity in cardiovascular deaths (48). Whether endothelial function and/or inflammatory response are improved and whether they are associated with the favorable outcomes in these trials remain to be determined.

Since positive cardiovascular outcomes have been observed in patients with established CVD by the use of anti-inflammatory agents [e.g., canakinumab (64)], interfering with AT inflammation could generate favorable outcomes. Indeed, in rodent models of obesity, several pharmaceuticals [e.g., metformin (49), resveratrol (50), and methotrexate (51)] are reported to reduce pro-inflammatory cytokine expression in AT and promote adiponectin expression, thereby rescuing eNOS phosphorylation and endothelial function.

Lifestyle intervention (e.g., exercise and diet) is one of the best approaches, especially when medicines are not available or existing medicines have failed. ED directly impairs basic vascular function (e.g., blood flow alteration), which made it a great target for pharmacological and/or exercise intervention with insulin-based therapies (57). Obese individuals who performed an acute high-intensity interval exercise presented with improved plasma pentraxin 3 and endothelial function (52). Even a short-term weight loss could reverse obesity-induced microvascular ED (53). Calorie restriction improved vascular insulin sensitivity, which was associated with downregulation of pro-inflammatory cytokine production in aged AT (54).

Given the functional AT-EC interaction, targeting both AT and EC (“dual-action therapies”) would be a better approach for obesity (65–67), which might lead to the cardiovascular benefits observed in different large-scale clinical trials (68).

## Discussion: Unresolved Questions and Future Directions

**Figure 1:**
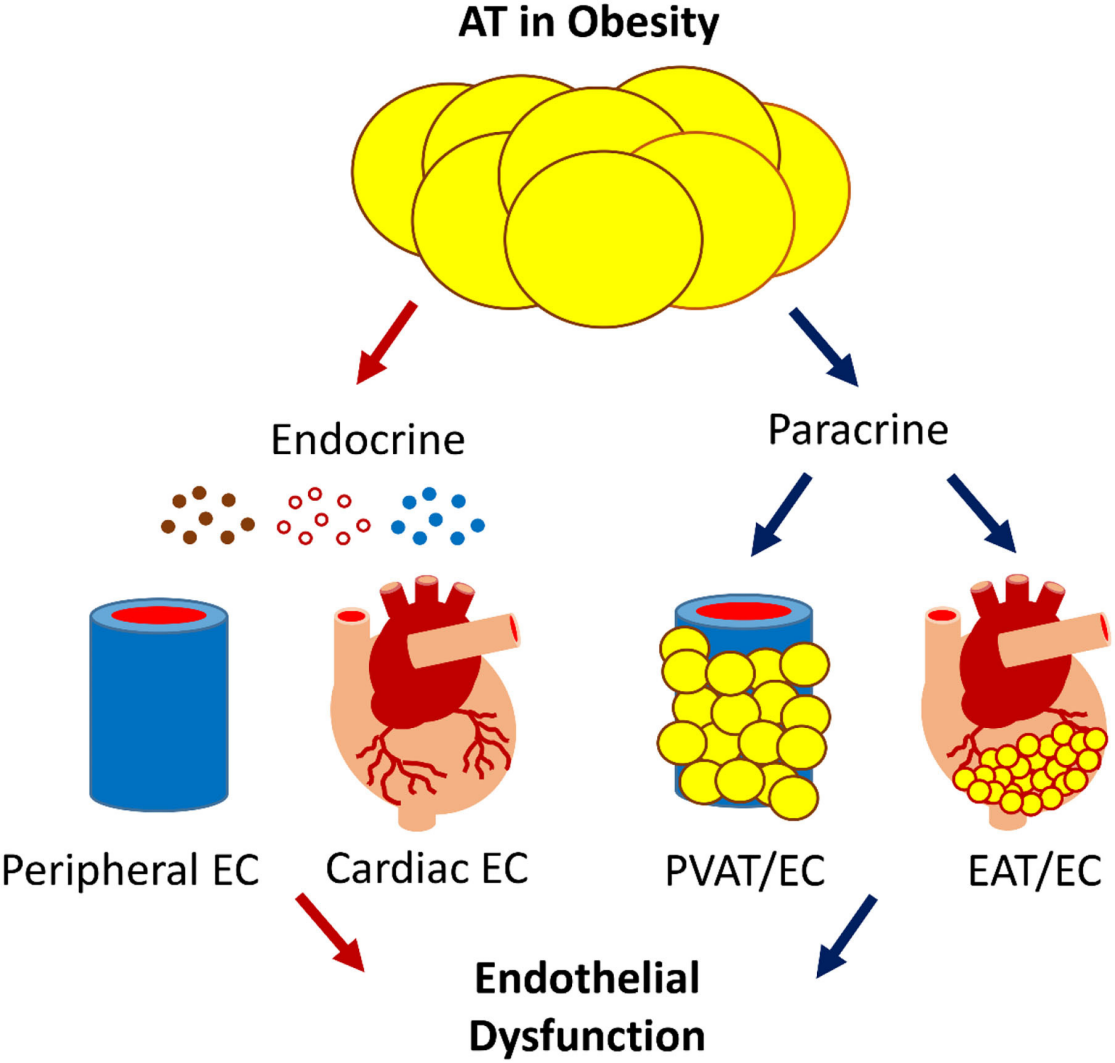
The scheme of AT-EC interplay in obesity-induced ED. AT interacts with the cardiovascular system via endocrine and paracrine secretion of bioactive products, e.g., adipokines, gaseous messengers, and microvesicles that carry bioactive molecules such as miRNA. Dysfunctional AT-EC interactions may induce ED in obesity, leading to CVD.

AT-induced ED is central to the development of CVD, the major cause of morbidity and mortality (69). In obesity, AT induces ED by releasing bioactive products locally and systematically (Figure 1). ED is the very first step in CVD pathogenesis; understanding the molecular mechanism could help us to identify therapeutic targets. Progress has been made in this regard, but important questions remain unanswered.

AT has striking biological variability due to its location and metabolic state, affecting the individual's overall cardiometabolic risk (70–73). For example, excess visceral AT has been linked to diabetogenic/atherogenic metabolic abnormalities more so than subcutaneous AT (73), partly because the former has more glucocorticoid receptors, which accelerated fat deposition when the hypothalamic-pituitary-adrenal axis was activated, leading to insulin resistance in the liver and in the skeletal muscle. To uncover how AT promotes CVD in obesity, we should consider both AT- expansion and its heterogeneous nature as an endocrine organ (74–78).

Large-scale epidemiological studies have questioned the exact nature of adiposity-adverse outcomes association, implicating an “obesity paradox,” in which individuals with overweight and even obesity present survival benefit compared with their normal-weight counterparts in general population and those with chronic diseases (79, 80) or critical illness (e.g., heart failure) (74–78). Although under debate (81), the survival benefits may be attributable to higher energy reserves, inflammatory preconditioning, endotoxin neutralization, adrenal steroid synthesis, activation of RAS, secretion of cardioprotective factors, and prevention of muscle wasting (82). Given the methodological flaws in these studies, further randomized and controlled clinical trials and prospective studies are required to validate the concept. Future research should focus on the pathophysiologic role of AT in critical illness. In this regard, the role and mechanism of endothelial (dys)function in the obesity paradox (83) remains to be elucidated.

Recent studies demonstrated that vascular-derived and heart-derived signals, such as pro-inflammatory and oxidative stimuli released from diseased vessels and/or myocardium, modified AT biology, e.g., by providing adipocyte precursors and driving angiogenesis in response to excess calories (84). Thus, AT can be regulated by feedback signals from the vascular EC, suggestive of a bidirectional interaction. Similar to EC control of CVD development (e.g., atherogenesis) by interacting with VSMC (85), emerging studies support that EC controls whole-body metabolisms through interactions with metabolic tissues (84), including AT in obesity-associated insulin resistance. The molecular mechanisms for bidirectional regulation of AT and EC merit continued investigation to better translate findings into clinical benefits.

The vasculature is present in all major organs, sustaining homeostasis and function throughout the body. The vascular EC display extensive functional heterogeneity depending on the vessel and tissue in which they reside. It facilitates the unique physiological function of each organ, such as nutrient transport, endocrine signaling, waste disposal, and disease protection. The mechanisms sustaining EC heterogeneity remain unknown (86). EC functional diversity was initially investigated by exploring EC specialization on a global scale [e.g., expression profile of multiple cultured EC with DNA microarrays (87)], followed by attempts to decipher functional and transcriptomic features of organ-specific EC in small populations or seldom-expressed genes in the lung (88), liver (89), heart (90), and other tissues (91). The emerging single-cell RNA sequencing technologies facilitate finding genes and pathways that dictate the organ-specific function of EC (92). A transcriptome study identified distinct gene expression profiles in cardiac EC (when compared with renal, cerebral, or pulmonary EC), e.g., higher expression of CD36 signaling cascade (90), which is a key regulator of fatty acid uptake and involved in atherogenesis (93). A recent study reported a relationship of circulating EC with obesity and cardiometabolic risk factors (94). Future research could identify signatures of the EC in depot-specific AT to determine their pathological roles in vascular complications.

The current COVID-19 pandemic presents an urgent health crisis (95). Numerous studies reported that severe obesity is associated with increased morbidity and mortality from COVID-19 (96–103), suggesting obesity as a risk factor for severe COVID-19 disease (104, 105). This is not surprising given that obesity is generally associated with increased incidence and severity of respiratory viral infection (106, 107). However, the underlying mechanism has yet to be elucidated. Recently, COVID-19 has been suggested as a multiorgan endothelial disease for its association with vasculitis and ED (108–111), which may be gender- and age-dependent (112). Although both the hypothetical role and therapeutic targetability of the vasculature in COVID-19 remain to be validated (113), an urgent need in this pandemic is to identify factors that mediate the physiological interactions between obesity and vasculature that contribute to CVD. Epithelial cell-derived IL-33 (114) was a key player in driving all stages of COVID-19 disease (115). EC also express IL-33 (116), the expression of which was enhanced in AT-EC by severe obesity (117). It would be timely to test whether EC-derived IL-33 mediates COVID-19-associated vascular complications (118). The hope is to bring about clinical breakthroughs for the treatment of COVID-19 in patients with obesity.

Obesity is a major risk factor for common medical conditions beyond CVD, such as type 2 diabetes (119), dyslipidemias (120), fatty liver (121), Alzheimer's disease (122, 123), and some cancers (124). These conditions occur due to obesity-induced insulin resistance and AT-derived endocrine factors (5). Given the essential roles of EC in the development of these disorders individually [diabetes (14), dyslipidemias, fatty liver (125, 126), Alzheimer's disease (127, 128), and cancers (129)], one would wonder whether targeting the AT-EC axis would be a novel avenue to improve these common conditions. Answers to these questions could be clinically significant in preventing or treating obesity-related complications.

## Author Contributions

JX proposed the conception. ML, MQ, KK, and JX wrote the article. All authors contributed to the article and approved the submitted version.

## Conflict of Interest

The authors declare that the research was conducted in the absence of any commercial or financial relationships that could be construed as a potential conflict of interest.
